# Membrane lipid raft homeostasis is directly linked to neurodegeneration

**DOI:** 10.1042/EBC20210026

**Published:** 2021-12-22

**Authors:** Tobias Moll, Jack N.G. Marshall, Nikita Soni, Sai Zhang, Johnathan Cooper-Knock, Pamela J. Shaw

**Affiliations:** 1 Sheffield Institute for Translational Neuroscience (SITraN), University of Sheffield, Sheffield, U.K.; 2 Department of Genetics, Stanford University School of Medicine, Stanford, CA, U.S.A.; 3 Center for Genomics and Personalized Medicine, Stanford University School of Medicine, Stanford, CA, U.S.A.

**Keywords:** apoptosis, enzyme activity, genetics, membrane dynamics, neurodegeneration, neurotrophic factors

## Abstract

Age-associated neurodegenerative diseases such as amyotrophic lateral sclerosis (ALS), Parkinson's disease (PD) and Alzheimer's disease (AD) are an unmet health need, with significant economic and societal implications, and an ever-increasing prevalence. Membrane lipid rafts (MLRs) are specialised plasma membrane microdomains that provide a platform for intracellular trafficking and signal transduction, particularly within neurons. Dysregulation of MLRs leads to disruption of neurotrophic signalling and excessive apoptosis which mirrors the final common pathway for neuronal death in ALS, PD and AD. Sphingomyelinase (SMase) and phospholipase (PL) enzymes process components of MLRs and therefore play central roles in MLR homeostasis and in neurotrophic signalling. We review the literature linking SMase and PL enzymes to ALS, AD and PD with particular attention to attractive therapeutic targets, where functional manipulation has been successful in preclinical studies. We propose that dysfunction of these enzymes is upstream in the pathogenesis of neurodegenerative diseases and to support this we provide new evidence that ALS risk genes are enriched with genes involved in ceramide metabolism (*P*=0.019, OR = 2.54, Fisher exact test). Ceramide is a product of SMase action upon sphingomyelin within MLRs, and it also has a role as a second messenger in intracellular signalling pathways important for neuronal survival. Genetic risk is necessarily upstream in a late age of onset disease such as ALS. We propose that manipulation of MLR structure and function should be a focus of future translational research seeking to ameliorate neurodegenerative disorders.

## Introduction

Membrane lipid rafts (MLRs) are specialised plasma membrane microdomains that are integral to the regulation of intracellular trafficking and signal transduction. MLRs contain high concentrations of four lipid classes: cholesterol, gangliosides, phospholipids and sphingolipids of which sphingomyelin is a subtype. In the brain, MLRs are present in neurons, astrocytes and microglia [1]. MLRs exist in two forms: either as flask-like invaginations termed caveolae (‘little caves’) or in a planar non-caveolar form [2]. In neurons, MLRs exist exclusively in the planar form and preferentially accumulate on somatic and axonal membranes. Neuronal MLRs are crucial for diverse functionality including neuronal adhesion, neuritogenesis, growth cone advancement, synapse formation and synapse maintenance [3–5]. In particular, MLRs modulate cell surface receptors that are positioned within their substance [6]. Consequently, pro-survival and pro-growth neuronal signalling pathways depend on the unique composition and regulation of lipids within the MLR, so much so that the majority of neurodegenerative diseases are linked with alterations in the biophysical properties of these microdomains [7,8]. Structural remodelling of MLRs is regulated by lipid-modifying enzymes [9]; certain types of these enzymes have been linked to neurodegeneration [10].

Neurotrophins are polypeptides that activate signalling pathways to promote the survival, development and function of neurons. The four most researched neurotrophins in mammals include nerve growth factor (NGF), brain-derived neurotrophic factor (BDNF), neurotrophin-3 (NT-3) and neurotrophin-4 (NT-4). Each of these neurotrophins can bind specifically to a tyrosine kinase (Trk) receptor, and all can bind to the low affinity p75 receptor [11], thus activating a downstream signalling cascade to drive the expression of pro-survival, pro-growth or pro-apoptotic genes [12]. These receptors are localised within MLRs and thus MLRs provide an essential platform for neurotrophin signalling pathways.

## MLR function is linked to neurodegenerative diseases

By definition neurodegenerative diseases are associated with ageing, and it is notable that ageing is associated with dysfunction directly and indirectly linked to MLRs. For example, age-associated loss of cholesterol from MLR leads to impaired pre-synaptic vesicle fusion and diminished neurotransmission [1,13]. Indeed ageing is associated with a generalised change in the composition of neuronal MLR including sphingomyelin [14] and gangliosides [15].

Amyotrophic lateral sclerosis (ALS) is an incurable age-of-onset neurodegenerative disorder affecting 1–2 per 100,000 people worldwide [16]. ALS is characterised by the progressive degeneration of upper and lower motor neurons within the motor cortex, brainstem and spinal cord, which leads to paralysis and death usually by respiratory failure within 2–5 years of symptom onset [17]. ALS is multifactorial with a number of described genetic and environmental risk factors. More than 50 potentially causative or disease-modifying genes have been associated with ALS; however, pathogenic variants in *SOD1*, *C9ORF72*, *FUS* and *TARDBP* are the most common [18]. Although phenotypically indistinguishable from one another, 10% of cases are familial, usually with an autosomal dominant inheritance pattern, whilst the remaining 90% are sporadic, defined as having no family history of the disease [17].

Neuronal cytoplasmic TDP-43-positive inclusions are the hallmark pathology of ALS which correlate with neuronal loss [19]. Disruption of MLRs is a feature of ALS pathophysiology [20,21] and is associated with impaired neurotrophic signalling [22]. Abnormal neurotrophic signalling is a feature of ALS [23,24], and in particular, deficient neurotrophic signalling associated with increased vulnerability to neuronal injury [25–27] is proposed as a mechanism underlying motor neuron toxicity. Neurotrophic signalling is an important regulator of apoptosis, and excessive neuronal apoptosis has also been observed in ALS [28].

Parkinson's disease (PD) is a debilitating neurodegenerative disorder of the motor system affecting 1–2 per 1000 people [29]. PD is characterised by the loss of dopaminergic neurons in the substantia nigra pars compacta [30]. The hallmark pathology of PD is the formation of α-synuclein aggregates within neurons in the form of Lewy bodies and Lewy neurites [31]. PD can affect multiple body systems, often as a consequence of autonomic nervous system dysfunction. Therefore, symptoms often include dysphagia, delayed gastric emptying and constipation [32]. Similar to ALS, alterations in the composition of MLRs have been reported in PD [33]. α-Synuclein has been shown to associate specifically with MLRs and this association is required for the normal synaptic localization of α-synuclein [34]. Excessive neuronal apoptosis has also been observed in PD [35].

Alzheimer's disease (AD) is a progressive neurodegenerative disorder and the most common cause of dementia. There are currently 40–50 million people living with dementia worldwide, and this number is expected to triple by 2050 [36]. AD is characterised by two hallmark pathologies in the brain: (i) the formation of extracellular amyloid beta (Aβ) plaques and (ii) deposits of neurofibrillary tangles of hyperphosphorylated tau protein [37]. These neuropathological changes are accompanied by glial cell activation, release of pro-inflammatory mediators and neuronal death, leading to brain atrophy [38]. Much like ALS and PD, MLRs are strongly linked with AD pathophysiology. BACE1 and γ-secretase are two enzymes involved in the cleavage of the amyloid precursor protein (APP) to produce Aβ. Both of these enzymes are targeted to MLRs [39,40], which increases the efficiency of processing of APP bound directly to cholesterol molecules within each lipid raft [41]. As a result, MLR structure directly promotes the production of Aβ plaques [42]. Indeed, disruption of MLRs [43] and excessive neuronal apoptosis [44] have previously been observed in AD. Moreover, interventions that preserve or restore MLRs can mitigate AD-associated neurodegeneration [45].

For the purposes of this review, two classes of lipid-modifying enzymes important for MLR homeostasis will be discussed: phospholipases (PLs) and sphingomyelinases (SMases). We aim to summarise evidence linking the function of PLs and SMases to neurodegeneration via MLR maintenance. We will focus on the archetypal neurodegenerative diseases ALS, PD and AD. Homeostasis of other lipid subtypes within MLRs including gangliosides and cholesterol have been reviewed elsewhere [10,46]. We suggest that dysregulation of PL and SMase enzymes can cause disruption within MLRs leading to altered neurotrophin-receptor binding; these enzymes produce second messengers which act downstream of neurotrophin receptors to modify signal transduction within the neuron. Impaired neurotrophic signalling is a well-described mechanism in the context of neurodegeneration [12].

## SMase dysregulation disrupts MLRs and impairs neurotrophic signalling

Sphingomyelin (SM) interacts with cholesterol to provide structural support for MLRs. Cholesterol is the ‘dynamic glue’ that holds MLRs together and most raft-associated proteins rely on cholesterol for their function [47]. SM expression is regulated by the sphingomyelin cycle where SM is hydrolysed to ceramide and phosphocholine by a small family of SMase enzymes; the reverse reaction is catalysed by SM synthase enzymes [48]. This mechanism is important for MLR homeostasis, but it also creates an environment to support efficient downstream neurotrophin signalling. SMases can be neutral (nSMase) or acidic (aSMase) depending on the optimal pH for their enzymatic activity [49]. Alterations in the activity of SMase enzymes operating within the sphingomyelin cycle have been frequently associated with neurodegeneration [50–52]. For example, inactivation of nSMase 2 correlates with the formation of TDP-43 aggregates in neuronal cells and exacerbates the disease phenotype in TDP-43 transgenic mice [53], and Aβ has been shown to directly activate SMase enzymes [54]. We argue that SMase dysregulation causes neurodegeneration via disruption of MLRs and a breakdown in the neurotrophic signalling cascade.

Ceramide is a product of SM metabolism that can act as a modulator of membrane structure or as a secondary messenger in intracellular signalling pathways [55] including signal transduction downstream of the p75 neurotrophin receptor [56–58]. Blocking ceramide production through inhibition of SMases can prevent NGF-induced apoptosis in hippocampal neurons after p75 activation i.e. downstream of receptor binding [59]. Ceramide also plays a key role in raft formation and growth [60,61] which has indirect consequences for activity of neurotrophin receptors including p75. Ceramide produced via activation of nSMases has been linked to neuronal apoptosis in SOD1-G93A ALS mice [62], and motor neurons over-expressing the ALS-associated SOD1-G93A protein are more susceptible to p75-induced apoptosis [63]. Given the complexity of this system, it is not surprising that the relationship between ceramide production and neurodegeneration is not linear: we have shown that ceramide can exacerbate neuronal toxicity. However, at low concentrations, ceramide can be neuroprotective and promote axonal development; for example, nSMase inhibition blocks the positive effects of the neurotrophic factor, NGF, on hippocampal neuron outgrowth [64].

Outside of p75 signalling, SMase activity has also been identified as a modulator of the other key neurotrophic signalling receptor subtype: Trk receptors. Binding of BDNF and NGF to Trk receptors promotes neuronal viability and is dependent on the presence of basal nSMase activity. Knockdown of nSMase in granule neurons and PC12 cells prevents neurotrophin-induced Akt phosphorylation [65]. Phosphorylation of Akt is an important rate-limiting step which protects cells from apoptosis and promotes cell survival [66].

nSMase enzymes have also been shown to influence the secretion of extracellular vesicles (EVs) from the plasma membrane [67,68]. Recently, a direct link between EVs and BDNF-dependent neurogenesis was described [69], which implicates nSMase enzymes in the regulation of neurotrophic signalling via modulation of EVs.

aSMase, encoded by the *SMPD1* gene, is a key enzyme involved in sphingolipid metabolism and regulation of MLR assembly [70]. MLR scaffolding proteins have been shown to directly interact with p75 and Trk receptors [71], and amplify TrkB signalling [22]. Dysregulation of aSMase has been shown to cause destabilisation of MLRs [72], which could lead to the impairment of downstream neurotrophin signalling. Reduced aSMase enzymatic activity has been described in PD patients with *SMPD1* mutations, and loss of aSMase expression correlates with increased α-synuclein levels *in vitro* [73]; indeed *SMPD1* mutations are associated with increased risk for PD [51]. We have previously demonstrated that coding and non-coding mutations which reduce function of caveolin-1 disrupt MLRs and increase the risk for ALS [74].

## Phospholipase dysregulation disrupts MLRs and impairs neurotrophic signalling

Phospholipases (PLs) catalyse the hydrolysis and cleavage of phospholipids [75] and therefore play a central role in plasma membrane structure and homeostasis which includes MLRs. Phospholipids form the membrane permeability barrier, but they also regulate membrane protein function and serve as second messengers in signal transduction pathways [76]. PLs consist of four large families (PLA, PLB, PLC and PLD), categorised by the position of the cleavage site on the phospholipid backbone [77]. As for SMases, PL activity is also an important source of secondary messengers and lipid signalling molecules, including free fatty acids, lysophospholipids, inositol 1,4,5-triphosphate (IP_3_), diacylglycerol (DAG) and phosphatidic acid (PA), which play an important role in neurotrophic signalling [78–80]. For example, altered expression of the second messenger atypical protein kinase C (aPKC), which is activated by phosphatidic acid that is generated by PLD [81], has been implicated in ALS [82].

Multiple studies have discovered that altered PL activity is associated with neurodegeneration [83–87] often via an effect on neurotrophic signalling. PLC-γ has been shown to be directly involved in regulating neurotrophic signalling, with roles in synaptic plasticity, neurite outgrowth, neurotransmission and neuronal excitability [88]. Similar to SMases, this activity is dependent upon p75 receptor signalling within MLRs. siRNA-mediated inhibition of PLC-γ activity abolishes p75 up-regulation stimulated by NGF [89]. Moreover, phosphatidylcholine-specific PLC (PC-PLC) induces apoptosis in response to glutamate toxicity in HT22 cells [90], which links MLR dysfunction and neurotrophic signalling to excitotoxicity. This is significant because excitotoxicity is proposed to be a central mechanism for ALS pathogenesis [91].

PL activity has been linked to other mechanisms associated with neurodegeneration although the link with MLRs is currently less clear. For example, studies in synaptic endings of rats have also shown that both PC-PLC and PLD1 promote increased activation of the lipid messenger, DAG, in response to iron-induced oxidative injury, highlighting a role for these lipid modifying enzymes in oxidative stress signalling [92,93]. cPLA2 signalling, via the activation of arachidonic acid (AA), mediates neuronal cell death in response to beta-amyloid peptide [94]. Finally, overexpression of wild-type α-synuclein in IMR-32 cells can cause inhibition of PLD1 signalling, leading to neurofilament loss and a reduction in cell viability [95].

**Figure 1:**
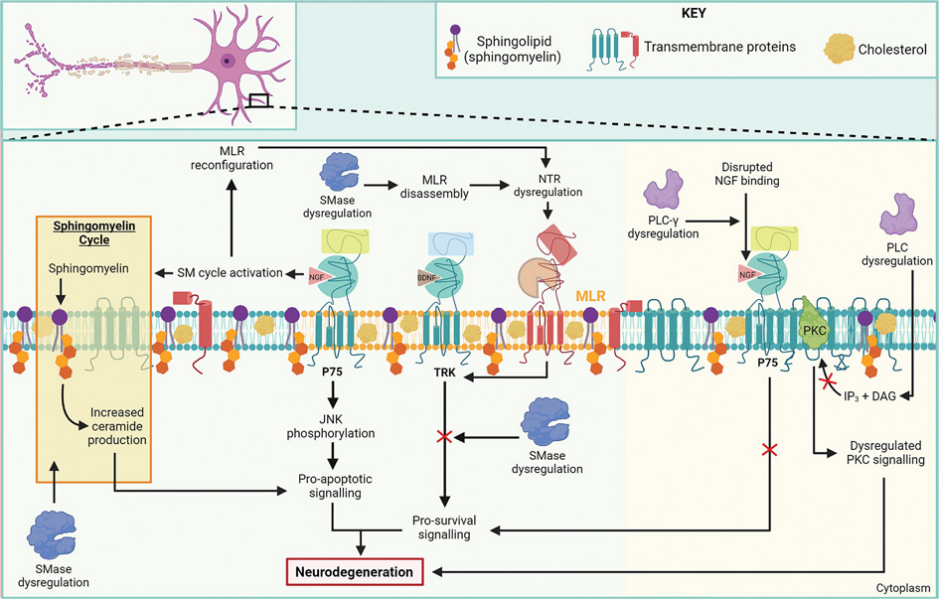
Neurodegenerative mechanisms associated with SMase and PL enzyme dysfunction converge on neurotrophin-regulated signalling pathways SMase enzymes have functional roles in the assembly of MLRs and the production of ceramide via the SM cycle. These processes are directly linked to different neurotrophin-regulated signalling pathways: MLRs promote TrkB signalling and ceramide influences NGF signalling via p75. SMase-mediated dysregulation of pro-survival signalling and increased pro-apoptotic signalling leads to neurodegeneration. Dysregulation of phospholipase C-γ prevents NGF-stimulated up-regulation of p75, leading to reduced axonal survival and growth. PLC dysregulation disrupts PKC signalling leading to neurodegeneration; DAG, diacylglycerol; IP_3_, inositol trisphosphate; JNK, c-Jun N-terminal kinase; NGF, nerve growth factor; NTR, neurotrophin receptor; PLC, phospholipase C; PKC, protein kinase C; SM, sphingomyelin; SMase, sphingomyelinase; TRK, tropomyosin-related kinase receptor.

Based on the biochemical, molecular and cellular evidence we have presented, we propose that SMase and PL enzymes play a central role in lipid biology and MLR homeostasis in particular, with consequences for neurotrophic signalling and neurodegeneration. A summary of the pathophysiological mechanisms linked to enzymatic dysregulation of SMases and PLs is provided in Figure 1.

## SMase and PL enzymes are promising therapeutic targets

Given their prominent role in apoptosis and neurotrophic signalling pathways, SMase and PL enzymes have been explored as potential therapeutic targets for ALS, PD and AD. Despite promising results, the selective targeting of SMase and PL enzymes is still in the preclinical stage. We have already described the complexity of this system demonstrating that effective neuroprotection may require up-regulation or down-regulation depending on specific biological context. However, given that overactivation of SMase and PL enzymes are most often associated with neurodegeneration, therapeutic targeting tends to focus on down-regulation of enzyme activity (Table 1). The attraction of targeting MLRs rather than neurotrophic signalling directly is that physiological control is maintained which should reduce the potential for excessive signalling leading to uncontrolled growth and potentially carcinogenesis.

**Table 1:** SMase and PL enzymes are potential therapeutic targets in neurodegenerative disease

Enzyme	Disorder	Disease associated change	Therapeutic target/Intervention
*Phospholipases*			
cPLA2	ALS	Increased mRNA expression in familial ALS patients and in ALS mice [107]	COX-2 inhibition reduced cPLA2 immunoreactivity and preserves motor neurons in the spinal cord of SOD1-G93A transgenic mice [107]
	AD	Increased activation in neurons [97]	Genetic ablation of cPLA2 prevents cognitive decline and preserves neuronal integrity in AD mice [108]
	PD	Increased expression in microglia [109]	cPLA2 -/- mice are more resistant to PD-associated neurotoxicity [110,111]
cPLA2α	ALS	Increased protein levels in sporadic ALS patients and in ALS mice [84,104]	CNS-specific reduction of cPLA2α reverses AD and ALS disease progression [104,112]
	AD	Increased immunoreactivity of cPLA2α and its transcript [113–115]	
PLCD1	ALS	Increased gene expression of PLCD1 and increased protein levels of PLCδ1 in ALS mouse model [100]	Genetic ablation of PLCD1 improves survival in ALS mice [100]
*Sphingomyelinases*			
aSMase	AD	Increased activity [50,97,116]	Genetic inhibition of aSMase ameliorates autophagic dysfunction in AD mice [50,99].
nSMase	AD	Increased activity [97]	Inhibition of nSMase decreases apoptosis and Aβ-induced cytotoxicity [97]

Enzyme expression changes, alterations in activity, associated mechanisms, associated neurodegenerative disorder(s), potential as therapeutic target, and references are shown; AD, Alzheimer's disease; ALS, amyotrophic lateral sclerosis; PD, Parkinson's disease.

### Sphingomyelinases

In theory, aSMase inhibitors could be used to prevent ceramide-driven apoptosis in neurodegenerative disease. The therapeutic potential of SMase inhibitors has not been investigated in pre-clinical models of ALS and PD. Although, indirect alteration of SMase function through modulation of sphingosine-1-phosphate, a known inhibitor of SMase enzymes [96], is currently undergoing phase II clinical trials for the treatment of ALS (Fingolimod (FTY720); NCT01786174). Conversely, SMase inhibition has been actively explored in AD [70]. SMase inhibition reduces the production of Aβ [54,97], and SMase inhibitors such as GW4896 and 3-O-methylsphingomyelin have been proposed as potential AD therapies [98], but translation to therapeutic trials has been limited by failure to achieve efficient target engagement [70]. Functional inhibitors of aSMase enzymes (FIASMAs) are a large group of compounds licensed for medical use which could have significant potential in neurodegenerative disease [99].

### Phospholipases

Knockdown of phospholipase C delta 1 (*PLCD1*), which encodes PLCδ1, in SOD1-G93A ALS mice delays symptom onset and prolongs survival [100]. This study also assessed nuclear shrinkage, a morphological change that occurs during apoptosis [100–102]. They found that motor neurons of PLCδ1^−/−^SOD1-G93A mice exhibited a reduction in nuclear shrinkage which would be consistent with an effect on neurotrophic signalling [100].

Cyclooxygenase-2 (COX-2) inhibitors have been shown to improve survival in the SOD1-G93A ALS mouse model [103] and to reduce expression of cytosolic PLA2 (cPLA2). Similarly, an antisense oligonucleotide against cPLA2α administered to SOD1-G93A mice ameliorates disease-associated elevation in levels of cPL2Aα protein, delays symptom onset and prolongs survival while preventing motor neuron loss [104].

## Genes involved in MLR homeostasis are associated with neurodegeneration

We have recently developed a novel machine learning model called RefMap to increase the power of gene discovery by integrating motor neuron functional genomics with ALS genetics [105]. We examined the 690 candidate genes identified by RefMap to see which are linked with MLR homeostasis and SMase/PL function in particular. Twenty-two genes that met the filtering criteria include: *ABCA1, ABCA2, ACER2, ANXA1, CERS5, GBA2, GRAMD1B, LPAR1, NAPEPLD, NTRK2, PLAA, PLPP6, S1PR3, SCARB1, SIGMAR1, SLC44A1, SPTAN1, TESK1, TMEM8B, TOR1A, TOR1B, TPD52*. Information regarding their functional role in MLR homeostasis along with previous associations with neurodegenerative disease is provided in Table 2. We tested for enrichment of GO pathways associated with SMase, PL and MLR function within RefMap ALS genes and discovered that ‘ceramide metabolic process’ (GO:0006672) genes are significantly enriched within RefMap ALS genes (*P*=0.019, OR = 2.54, Fisher exact test). As a background we assumed the total set of genes expressed in iPSC-derived motor neurons (TPM>1, *n*=19,516) [89]. Overall, this suggests that dysfunction of MLR homeostasis and ceramide metabolism in particular may be upstream in the pathogenesis of ALS.

**Table 2:** MLR-associated ALS genes identified using RefMap

RefMap ALS gene	Functional role in MLR homeostasis	Associated neurodegenerative disease	References
ABCA1	Reduces formation of lipid raft domains	ALS	[117]
ABCA2	Regulates cholesterol and ceramide homeostasis	ALS, AD	[118]
ACER2	Catalyses the hydrolysis of ceramides to generate sphingosine	ALS	[119]
ANXA1	Facilitates MLR clustering; inhibitor of phospholipase A2	ALS	[120]
CERS5	Synthesises c-16 ceramide, which is a pro-apoptotic ceramide	ALS	[121]
GBA2	Catalyses the conversion of glucosylceramide to free glucose and ceramide	ALS, HSP	[122]
GRAMD1B	Regulates membrane cholesterol homeostasis	ALS	[123]
LPAR1	G-protein coupled receptor that localises to MLR	ALS, PD, AD	[124,125]
NAPEPLD	A phospholipase D that cleaves N-acylphosphatidylethanolamines (NAPEs). NAPEs are involved in the consolidation of MLR structure	ALS, PD	[126,127]
NTRK2	Encodes neurotrophic tyrosine kinase receptor type 2, enriched within MLRs	ALS, AD	[128]
PLAA	Phospholipase A2-activating protein present within MLRs; phospholipase A2 hydrolyses membrane phospholipids	ALS	[129]
PLPP6	Phospholipid phosphatase, involved in cholesterol synthesis	ALS	[130]
S1PR3	Sphingosine-1-phosphate receptor 3; sphingosine 1-phosphate (S1P) is a bioactive phospholipid growth factor that recruits proteins to MLRs	ALS	[131]
SCARB1	Localises to MLR and caveolin-1, involved in cholesterol homeostasis	ALS, AD	[132]
SIGMAR1	ER-resident protein with roles in MLR homeostasis	ALS, AD	[133–136]
SLC44A1	Encodes choline transporter-like protein 1, involved in phospholipid synthesis and MLR homeostasis	ALS	[137]
SPTAN1	Spectrin alpha chain, non-erythrocytic 1, associates with MLRs	ALS, HSP, PD, AD, ataxia	[138–140]
TESK1	Interacts with Spred1 which localizes in lipid raft/caveolae and inhibits ERK activation in collaboration with caveolin-1	ALS	[141]
TMEM8B	May have phospholipase A2 activity	ALS	[142]
TOR1A / TOR1B	Encodes member of torsin protein family: Torsins are MLR-associated proteins important for lipid metabolism	ALS	[143]
TPD52	Interacts with the lipid raft protein MAL2	ALS	[144]

RefMap ALS gene ID, role within MLR homeostasis, associated neurodegenerative disorder(s) and references are shown; AD, Alzheimer's disease; ALS, amyotrophic lateral sclerosis; HSP, hereditary spastic paraplegia; PD, Parkinson's disease.

## Conclusion and future directions

MLRs represent an organising centre for neurotrophic signalling in neurons. MLR disruption has already been described as an upstream cause of neurodegeneration [74]. Homeostasis of MLRs is tightly coupled to downstream signalling, particularly for Trk and p75 neurotrophin receptors which are positioned within the MLR. We have focused on SMases and PLs showing how activities of these enzymes are crucial modulators of neurotrophic signalling. Moreover, we have summarised evidence from the literature demonstrating that over activity of SMase and PL enzymes is linked to abnormal neurotrophic signalling and ultimately excessive apoptosis and neurodegeneration. A large number of studies provide specific examples linked to ALS, AD and PD.

An open question remains regarding the position of MLR dysfunction in the cascade of pathophysiology leading to neurodegenerative disease. It is possible that dysfunction of MLRs is simply a downstream consequence of neuronal toxicity. However, several arguments suggest that the role of SMases and PLs are more significant than that: firstly, these enzymes and their products have been linked to key mechanisms associated with these diseases including excitotoxicity, oxidative stress and protein aggregation. Second, manipulation of these enzymes can ameliorate the phenotype in key models of neurodegeneration. Finally, we show here evidence that proteins in these pathways are enriched with genetic mutations linked to ALS. Genetics are largely fixed at conception and so are, by definition, upstream in the cascade of pathogenesis leading to a late age of onset disease.

In this review we have focused on ALS, PD and AD, which are the most frequent neurodegenerative diseases. However, we acknowledge that this is far from an exhaustive list and although the evidence-base is strongest for these conditions, it is likely that other diseases are not represented only because key experiments have not yet been performed. In particular, FTD is on a spectrum with ALS and both diseases share key pathogenic mechanisms [106]; we suspect that much of the evidence we have presented for ALS would also apply to FTD.

We suggest that MLRs should be a key focus of translational research for neurodegenerative diseases. The potential is a means of manipulating neurotrophic signalling in a controlled and specific manner, which does not bypass physiological production of neurotrophins. Manipulation of SMases and PLs could improve pro-survival signalling efficiency by efficient organisation of neurotrophin receptors within the MLR, and simultaneously modulate second messenger transduction following receptor binding. The field awaits further validation using patient-derived cell models which are noticeably missing from the literature currently, to address this important opportunity.

## Summary

- Membrane lipid rafts (MLRs) are essential signalling platforms, and their dysregulation is strongly linked with neurodegenerative disease.
- MLR homeostasis is regulated by sphingomyelinase (SMase) and phospholipase (PL) enzymes, and functional changes in these enzymes can cause impaired neurotrophic signalling and excessive apoptosis.
- Genes involved in MLR homeostasis are enriched with genetic risk for amyotrophic lateral sclerosis (ALS).
- SMase and PL enzymes are promising therapeutic targets for the treatment of neurodegenerative diseases.

## Abbreviations

AD: Alzheimer's disease
ALS: amyotrophic lateral sclerosis
DAG: diacylglycerol
IP_3_: inositol trisphosphate
JNK: c-Jun N-terminal kinase
NAPE: N-acylphosphatidylethanolamine
NGF: nerve growth factor
NTR: neurotrophin receptor
PD: Parkinson's disease
PL: phospholipase
PLC: phospholipase C
PKC: protein kinase C
MLR: membrane lipid raft
SM: sphingomyelin
SMase: sphingomyelinase
TRK: tropomyosin-related kinase receptor
